# Genome-informed qPCR tracking revealed preferential persistence of *Bacillus subtilis* BS9 in the broiler chicken gastrointestinal tract

**DOI:** 10.1128/spectrum.00926-26

**Published:** 2026-09-17

**Authors:** Junli Ni, Haiming Cai, Alison Gelinas, Xiaoshu Zhan, Nanshan Qi, Elijah G. Kiarie, Julang Li

**Affiliations:** 1 Institute of Animal Health, Guangdong Academy of Agricultural Sciences 117866, Guangzhou, Guangdong, China; 2 Department of Animal Biosciences, University of Guelph 3653, Guelph, Ontario, Canada; 3 School of Animal Science and Technology, Foshan University 47868, Foshan, Guangdong, China; Instituto Nacional de Tecnologia Agropecuaria, Buenos Aires, Argentina

**Keywords:** *Bacillus subtilis*, persistence, chicken intestinal tract, qPCR-based specific detection

## Abstract

This study aimed to develop a strain-specific quantitative PCR (qPCR) assay for *Bacillus subtilis* BS9 and characterize its persistence and spatial distribution in the broiler chicken gastrointestinal tract. Whole-genome sequencing and comparative genomic analysis identified a unique 110-bp sequence within a strain-specific genomic island, which was used to design a highly specific qPCR assay with excellent efficiency and sensitivity. In a 14-day *in vivo* trial, broiler chicks receiving daily oral doses of BS9 were analyzed using both culture-based methods and the newly developed qPCR. The assay was applied qualitatively, presence or absence, to detect BS9 in intestinal samples. BS9 was detected exclusively in the duodenum, jejunum, and cecum, with no presence in the gizzard or ileum. These findings demonstrate that BS9 exhibits region-specific persistence in the gut, likely reflecting adaptation to distinct physiological niches, which may contribute to its probiotic mechanisms.

**IMPORTANCE:**

This work provides the first detailed account of *B. subtilis* BS9’s spatial persistence in poultry, revealing preferential adherence to specific intestinal regions. The strain-specific qPCR assay developed here offers a precise, culture-independent tool for tracking BS9 in complex gut environments. These insights into the genetic basis and tissue tropism of BS9 persistence advance our understanding of probiotic-host interactions and establish a framework for characterizing novel probiotic strains.

## INTRODUCTION

The global livestock industry has been facing issues related to antibiotic misuse and residues, leading to the implementation of regulations promoting antibiotic reduction, prohibition, and substitution (1). This has made antibiotic-free animal agriculture a goal in animal production. Research has shown that probiotics can modulate the gut microbiota, stimulate the immune system, mitigate inflammatory responses, prevent pathogen colonization, enhance growth performance, and reduce ammonia and urea excretion, reviewed in reference 2. Therefore, the development of more efficient probiotics is of critical importance for preventing pathogenic infections in broilers and advancing strategies for antibiotic replacement.

*B. subtilis* is a Gram-positive, spore-forming bacterium exhibiting aerobic or facultative anaerobic metabolism. It is commonly administered in the form of spores as a feed additive for animals. Following ingestion, the spores undergo germination and proliferate within the gastrointestinal tract, where they exert their probiotic functions. In terms of immunomodulation, *B. subtilis* exhibits bidirectional regulatory effects. Its bacterial cells and metabolites not only act as immunostimulants to enhance non-specific immunity but also modulate relevant signaling pathways to alleviate intestinal inflammation (3). Within the intestinal environment, *B. subtilis* is capable of secreting a spectrum of digestive enzymes, including proteases, amylases, lipases, and non-starch polysaccharidases, improving the efficiency of nutrient breakdown and absorption. Beyond digestive support, *B. subtilis* can also produce antioxidant enzymes, such as superoxide dismutase (SOD), that help mitigate oxidative damage to intestinal tissues and cells (4).

Our laboratory previously isolated and characterized a novel *B. subtilis* strain, BS9. It was found that this strain can survive in various bile salts and low pH environments. It exhibits significant bactericidal effects against enterotoxigenic *Escherichia coli* (ETEC), *Salmonella Typhimurium*, and methicillin-resistant *Staphylococcus aureus* (MRSA) through a contact inhibition mechanism, demonstrating strong antimicrobial properties (5). In a porcine intestinal cell (IPEC-J2) model, BS9 inhibited ETEC by reducing its attachment and attenuating the associated inflammatory and cytotoxic responses (6). Furthermore, our broiler trials revealed that BS9 improves production performance and alleviates intestinal symptoms in coccidia-infected chickens (7). In studies on weaned piglets, BS9 demonstrated the potential to replace zinc oxide via distinct mechanisms, enhancing feed utilization and production performance (8). These findings collectively underscore its promise as a probiotic feed additive for poultry and livestock.

In the complex intestinal environment, probiotics exert their functional properties by competitively colonizing the gut and inhibiting harmful bacteria, further enhanced through the production of beneficial compounds, such as antimicrobial peptides (AMPs), vitamins, and enzymes. Therefore, regardless of their specific mode of action, successful colonization is a desirable feature for probiotic efficacy. For most allochthonous (externally administered) probiotics, colonization is typically transient. Building on the model of *L. reuteri*, Walter et al. provided a detailed discussion on the colonization mechanisms and barriers for both commensal and probiotic bacteria, noting that exogenous probiotics struggle to achieve long-term colonization in an intestine with a stable resident microbiota (9). In studies where mice were administered various single- or multi-strain probiotics, Zmora et al. reported that mucosal colonization was highly individualized. Their findings indicated that the gut of most vertebrates resists probiotic colonization; probiotics are either completely excluded or only transiently persist. Successful colonization appears to be closely tied to host-specific features, including the native gut microbiota composition and host gene expression (10). Consequently, elucidating the duration and spatial distribution of the colonization of the probiotic is critical for understanding the mechanisms underlying their beneficial effects and serves as one of the indicators of the practical effectiveness of the probiotic (11). Our study thus aims to determine the spatial distribution and persistence of BS9 in the gastrointestinal tract of broiler chickens at 14 days post-administration, using a newly developed strain-specific qPCR assay to provide single time-point snapshot of BS9 persistence.

## MATERIALS AND METHODS

### Bacterial strain

The single strain *B. subtilis* (BSDFM), originally named *B. subtilis* CP-9, now renamed *B. subtilis* BS9, was isolated from camel feces and characterized at the University of Guelph (12).

### Genome sequencing, assembly, and annotation

Genomic DNA was purified using the Invitrogen Purelink Genomic DNA Mini Kit (K182001). Sequencing was performed on an Illumina MiSeq platform, at the Genome Sequencing Unit of the CGEB-Integrated Microbiome Resource, Dalhousie University (Canada), using a shotgun paired-end library according to standard protocols. The quality of the raw paired-end Illumina MiSeq reads was initially assessed using FastQC (v0.12.1). Subsequently, adapters and low-quality sequences were removed using fastp (v1.0.1) with its default parameters for quality filtering and adapter trimming. The resulting high-quality reads were utilized for *de novo* genome assembly with SPAdes (v4.0.0), employing the careful option to minimize mismatches and short indels. The quality and completeness of the final genome assembly were evaluated using QUAST (v5.3.0) against the reference genome of *B. subtilis* strain 168 (GenBank accession: NC_000964.3).

The assembled genome of *B. subtilis* BS9 was annotated using Prokka (v1.14.6). This pipeline identified protein-coding sequences (CDS) using Prodigal (v2.6.3), ribosomal RNA (rRNA) genes with Barrnap (v0.9), and transfer RNA (tRNA) genes with Aragorn (v1.2.41). Initial functional annotation of the predicted proteins was performed by searching against the bacterial protein databases integrated within the Prokka pipeline. The genome map of *B. subtilis* BS9 assembly, displaying contigs larger than 5,000 bp, gene features on both strands, GC content, and GC skew, was generated with the R package circ lize v0.4.16 in R 4.2.2 and RStudio 2022.07.2 (Build 576) software.

### Functional annotation and COG analysis

To obtain comprehensive functional information, the protein sequences predicted by Prokka were further annotated using eggNOG-mapper (v2.1.13). The analysis was conducted using the DIAMOND mapping mode (-m diamond) against the eggNOG 5.0.2 database. Key parameters included setting the taxonomic scope to Bacteria (--tax_scope 2), an e-value cutoff of 1e−3, and minimum alignment score and identity thresholds of 60 and 40%, respectively. For downstream functional analysis, genes were assigned to Clusters of Orthologous Groups (COG) categories, and their distribution was visualized as a bar chart generated with the R package ggplot2 v3.5.2.

### Comparative genomic analysis and visualization

To identify unique genomic regions in *B. subtilis* BS9, a whole-genome alignment was performed against the reference strain *B. subtilis* 168. The complete genome of the reference strain was retrieved from the NCBI database using E-utilities (v22.4). The alignment and identification of locally collinear blocks (LCBs) were carried out using progressiveMauve (snapshot_2015_02_13). Genomic regions present in *B. subtilis* BS9 but absent in the reference genome were identified as strain-specific. The R package Biostrings v2.66.0 was employed to pinpoint the precise coordinates of target marker sequences within the Mauve alignment backbone file, confirming their location within these specific regions. The gene organization within the primary identified unique region was visualized linearly using the R package gggenes v0.5.1 in RStudio, providing a clear basis for the design of strain-specific identification primers. To ensure the primers were specific not only against the reference strain 168 but also against a broader range of reported sequences, their specificity was further verified *in silico* using the NCBI Primer-BLAST tool. The results confirmed the absence of non-specific binding, validating the high specificity of the primers for *B. subtilis* BS9.

### Development and validation of a strain-specific qPCR assay

Based on the previously identified strain-specific gene, a pair of qPCR primers (S2-2) was designed to target its unique sequence: forward primer 5′-ATCAGCCGATCCGTTGTTGA-3′ and reverse primer 5′-CGCTGTTAATTTCAGCCGGT-3′ (Table S1). The qPCR protocol was established as follows: an initial denaturation at 95℃ for 30 s; followed by 40 cycles of denaturation at 95℃ for 15 s and a combined annealing/extension at 60℃ for 30 s with fluorescence acquisition. Finally, a melt curve analysis was performed from 65.0℃ to 95.0℃ with 0.5℃ increments held for 5 s each. A primer concentration gradient (10, 5, 2.5, 1.25, and 0.625 μM) was tested to determine the optimal amplification efficiency.

Standard curves were generated using genomic DNA extracted from serial dilutions of an overnight *B. subtilis* BS9 culture. CFU counts were determined by parallel plating on LB agar. Genomic DNA was extracted from the same dilutions using the Invitrogen Purelink Genomic DNA Mini Kit (K182001), and qPCR was performed to establish the relationship between Cq values and CFU equivalents. The standard curve ranged from 1.0 × 10⁷ to 1.0 × 10¹ CFU/mL.

Subsequently, serial dilutions of the template (ranging from 1.0 × 10^7^ to 1.0 × 10^3^ CFU/mL) were used to assess the sensitivity of the primers and calculate the amplification efficiency. Finally, the specificity of the primers was validated by testing against closely related bacterial strains and other common gut microbiota species, including *Escherichia coli* ATCC 25922, *Staphylococcus aureus* ATCC 6538, *Bacillus subtilis* ATCC 6051, *Bacillus subtilis* strain 168, as well as laboratory stocks *Bacillus megaterium*, L1 (*Bacillus subtilis*), L2 (*Bacillus cereus*), L5 (*Bacillus subtilis*) from lake mud, and B4 (*Bacillus licheniformis*), B5 (*Bacillus subtilis*) from camel feces (Table S2). The determination of the limit of detection (LOD) was based on the results of the no-template control (NTC). Using the Cq value from the template-free detection as a reference, the positive threshold was defined as exceeding the NTC Cq value by 3, corresponding to a Cq value of 35. Consequently, by integrating this threshold with the standard curve, the dilution factor corresponding to the LOD was determined.

### Animal trial

The production and preparation methods for BSDFM adhered to the procedures outlined by Sudan et al. (8) and detailed by Gelinas et al. (7). The final BSDFM stock was prepared to contain 1.0 × 10^8^ colony-forming units (CFU) of BSDFM/mL. For the animal trial, a total of 64 mL of fresh culture was prepared daily and divided into 32-mL tubes for morning and afternoon administration. The afternoon culture was stored in a refrigerator until application. Therefore, birds in the +BSDFM group received 2.0 × 10^8^ CFU/mL BSDFM daily.

**Fig 1:**
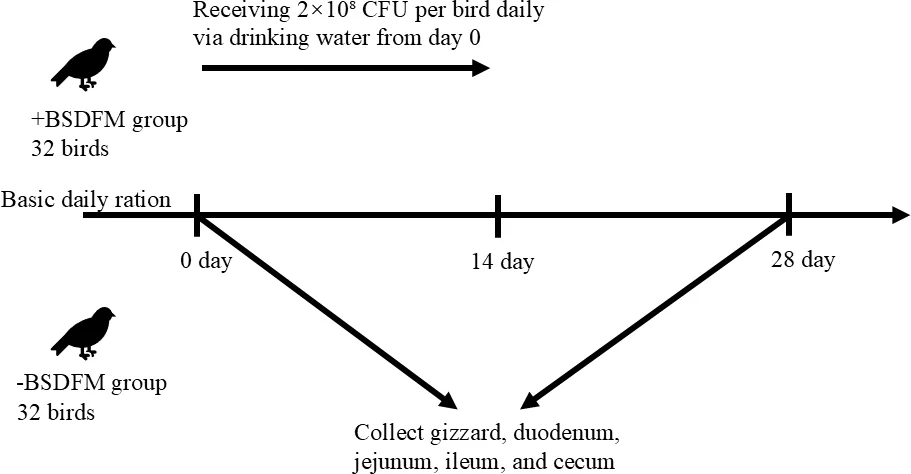
The animal experiment design drawing. +BSDFM group: From 0 to 14 days of age, each bird received 2 × 10⁸ CFU of BS9 daily and was fed a basal diet from 0 to 28 days of age. Samples of the gizzard, duodenum, jejunum, ileum, and cecum were collected at 0 and 28 days of age, respectively. −BSDFM group: The control group was fed a basal diet from 0 to 28 days of age. samples of the gizzard, duodenum, jejunum, ileum, and cecum were collected at 0 and 28 days of age, respectively.

The animal utilization protocol (#4403) for this experiment was approved by the University of Guelph Animal Care Committee, and all birds were cared for in accordance with the Canadian Council on Animal Care Guidelines (CCAC, 2009). As shown in Fig. 1, 66 1-day-old male broiler chickens were procured from a commercial hatchery (Maple Leaf Foods, New Hamburg, ON, Canada). Two birds were necropsied for sample collection on arrival at the Arkell Poultry Research Station (Guelph, ON). The remaining chicks were randomly assigned to two groups (*n* = 32/group): a control (−BSDFM) and a treatment (+BSDFM) group, housed in separate experimental rooms to minimize contamination. Gelinas et al. (7) describe the details of housing, biosecurity, husbandry, and management for broiler chickens. Both groups received a starter (d0–13) and grower (d14–28) basal diet formulated to meet or exceed specifications (7). The diets were prepared by the Arkell Feed Mill (Guelph, ON, Canada), consisting of a crumble for the starter and a pellet for the grower.

The application of BSDFM was similar to that described by Gelinas et al. (7), with the addition of sodium carboxymethyl cellulose (CMC) to drinking water. The calculated amount of water provided with the BSDFM differed from that of Gelinas et al. (7), as it was intended to be consumed within 45 min of provision, modified from methods by Kim et al. (13). The supplies for the treatment groups were separated, and the −BSDFM preparation occurred first to reduce cross-contamination. Briefly, 50 g of CMC was diluted with 2 L of MQ water and heated, stirring until dissolved to provide a 2.5% CMC solution, which was then divided into two 1-L flasks for use. On days 0–13, in the morning and evening, the water measured for the different life stages was added to separate clean beakers. The +BSDFM group received their 32mL BSDFM solution in their beaker or 1.0 × 10⁸ CFU of *B. subtilis* BS9. A syringe was then used to measure CMC to a target concentration of 0.5g/L in drinking water. The solutions were thoroughly mixed with a glass stirring stick. Water lines were raised 45 min before administration to drive thirst. The final solutions were added to the bell drinkers of their respective treatment group. After the solution was consumed, the drinkers were removed from pens, and the waterlines were lowered.

To evaluate the persistence of colonization, six birds per pen were euthanized by cervical dislocation and necropsied at 28 days of age (14 days post-BS9 administration), as described by Gelinas et al. (7). A small 1-cm piece was collected from the gizzard, duodenum, jejunum, ileum, and cecum. The tissue samples were put directly into a vial with reagent, immediately placed on ice and transported to the laboratory, where they were stored frozen at −80°C until analysis.

### Analysis of bacterial persistence

The six tissue samples from each intestinal segment were divided into two pools: samples 1–3 were pooled as Pool A, and samples 4–6 were pooled as Pool B. Genomic DNA was extracted from each pooled homogenate and analyzed using the strain-specific qPCR assay. Each pool was tested in three technical replicates. An intestinal segment was considered qPCR-positive when both Pool A and Pool B yielded positive amplification (Cq ≤ 35) in all three technical replicates.

An additional pooled sample was prepared by mixing equal amounts of all six individual samples (1–6) from each intestinal segment. This mixed sample was serially diluted, plated on LB agar, and incubated at 37°C for 12 h under aerobic conditions. Ten single colonies were randomly selected from LB agar plates streaked with tissue homogenates and suspended in ddH₂O. Strain-specific qPCR was performed using the pre-optimized reaction system to verify amplification specificity and melting curve profiles. An intestinal segment was considered positive for *B. subtilis* BS9 persistence only if both Pool A and Pool B yielded positive qPCR results in all three technical replicates, and at least one of the ten randomly picked colonies from the intestinal segment showed a specific qPCR signal with a quantification cycle (Cq) value ≤35.

### Statistical analysis

For each intestinal segment per group, direct qPCR was performed on two pooled samples (Pool A and Pool B), each with three technical replicates. A segment was scored positive only when both pools yielded consistent positive amplification across all replicates. Due to the pooling strategy that generated two independent samples per segment per group, detection outcomes are reported descriptively as positive or negative, with colony qPCR results (positive colonies/10 colonies tested) serving as confirmatory evidence. No inferential statistical testing was performed between groups, as the experimental design was optimized for the detection of BS9 presence rather than comparative prevalence estimation.

## RESULTS

### Genome assembly and general features

**Fig 2:**
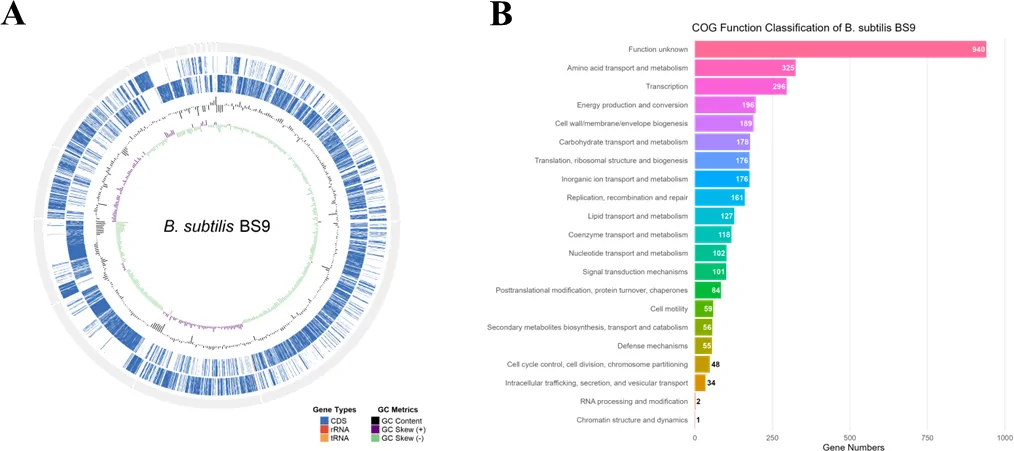
Genomic features and functional classification of *B. subtilis* BS9. (**A**) Circular genome map of the *B. subtilis* BS9 assembly. The tracks, from the outermost to the innermost circle, represent: CDS (blue), tRNA (orange), rRNA (purple), GC Content (black), GC skew positive (green), and GC skew negative (purple). (**B**) Functional classification of 4,057 predicted protein-coding genes based on clusters of orthologous group (COG) categories. The bars represent the number of genes assigned to each functional category.

The complete genome of *B. subtilis* BS9 was sequenced using the Illumina MiSeq platform and assembled *de novo*. The final assembly resulted in a genome of 3,973,582 bp with a GC content of 45.69%. The assembly consists of 54 total contigs, with 23 contigs being larger than 500 bp. The assembly quality was high, as indicated by an N50 value of 466,986 bp and an L50 of 3, meaning that 50% of the entire genome is contained in only three contigs. Genome annotation predicted a total of 4,057 coding sequences (CDS), 84 tRNA genes, 14 rRNA genes, and 1 tmRNA gene (summarized in Table 1). A circular map illustrating the overall architecture of the assembled genome, including the distribution of genes, GC content, and GC skew, is presented in Fig. 2A.

**TABLE 1:** General features of the *B. subtilis* BS9 genome

Genome	*B. subtilis* BS9
Size	3,973,582 bp
GC content	45.69%
Number of coding sequences	4,057
Number of tRNA	84
Number of rRNA	14
Number of tmRNA	1

### Functional annotation of the *B. subtilis* BS9 genome

To understand the functional potential of *B. subtilis* BS9, all predicted protein-coding genes were classified into COG categories. The distribution of these categories is shown in Fig. 2B. A significant portion of genes (940, ~23%) was assigned to “Function Unknown,” a common category in newly sequenced genomes. Among the genes with predicted functions, the most abundant categories were amino acid transport and metabolism (325 genes), transcription (296 genes), and energy production and conversion (196 genes). These results suggest that the *B. subtilis* BS9 genome contains a robust set of genes for core metabolic activities, biosynthesis, and genetic regulation, consistent with the lifestyle of *B. subtilis*.

### Comparative genomics and identification of a strain-specific marker

**Fig 3:**
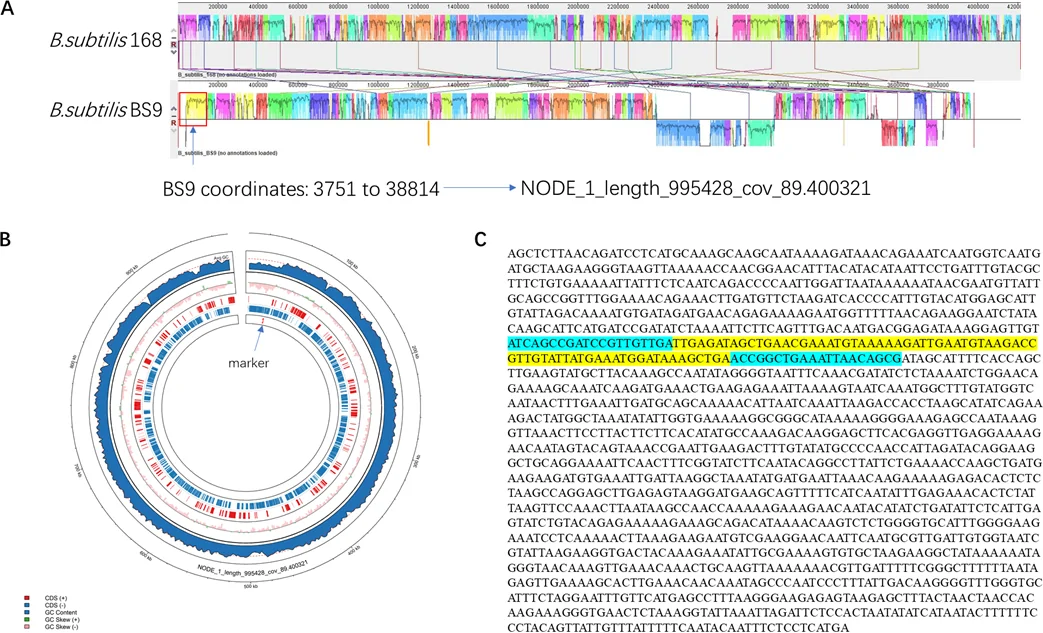
Identification of a strain-specific genomic region in *B. subtilis* BS9. (**A**) Whole-genome alignment of *B. subtilis* BS9 (Hevcox) against the reference strain *B. subtilis* 168 (cp) using Mauve. Colored locally collinear blocks (LCBs) indicate homologous regions shared between the genomes. The orange box highlights a large genomic region (coordinates 3751 to 35514 on NC001_1) that is present in BS9 but absent in strain 168. (**B**) Circular map of contig NC001_1_length_929528_cov_50.003321. The tracks, from the outermost to the innermost circle, represent CDS on the forward strand (red), CDS on the reverse strand (blue), GC content, and GC skew. The blue arrow indicates the location of the identified strain-specific marker region. (**C**) Nucleotide sequence of the target amplicon within the marker region. The forward primer sequence (5′-ATCAGCCGATCCGTTGTTGA-3′) and the reverse primer sequence (5′-CGCTGTTAATTTCAGGCCGGT-3′) are highlighted in cyan.

To identify unique genetic regions in *B. subtilis* BS9, its genome was aligned with the reference genome of *B. subtilis* strain 168 (Fig. 3A). The whole-genome alignment revealed several genomic regions present in *B. subtilis* BS9 but absent in the reference strain. A large, strain-specific genomic island of approximately 35 kb was identified within the largest contig, NODE_1_length_995428_cov_89.400321 (Fig. 3A, orange box). This unique region, highlighted in the circular map of the contig (Fig. 3B), serves as a potential target for developing a strain-specific molecular marker that is absent in all publicly available reference strains. Based on the sequence of this region, a pair of qPCR primers was further designed *in silico* using NCBI Primer-BLAST against the nucleotide collection (nr/nt) database, and designed to amplify a unique 110 bp fragment (Fig. 3C).

### Validation of the strain-specific qPCR assay

**Fig 4:**
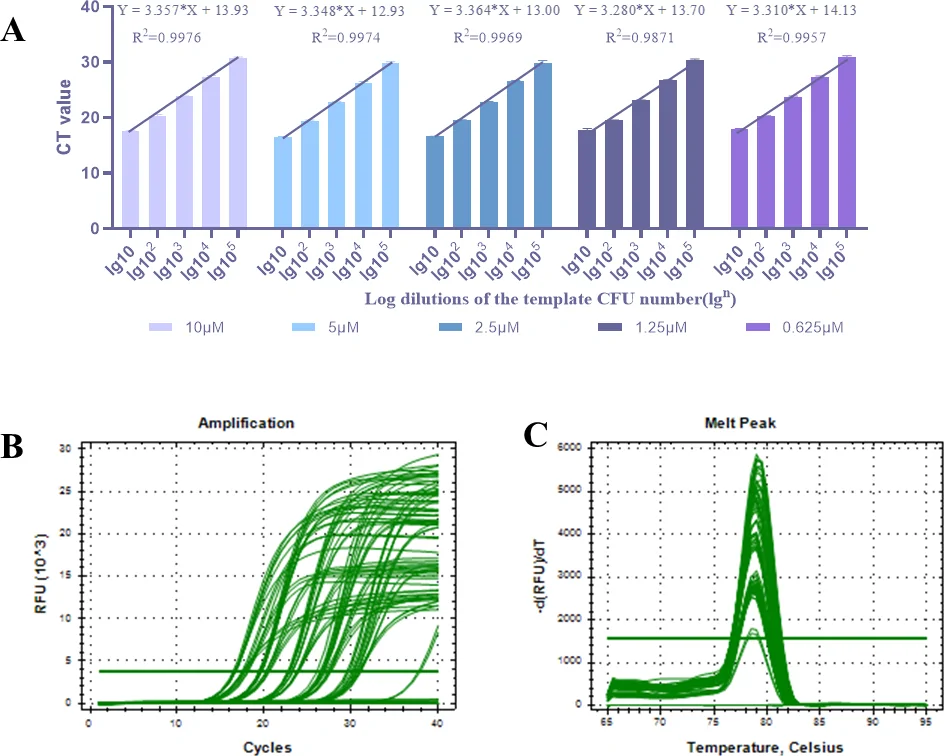
The amplification standard curve. (**A**) Standard amplification curves obtained with different primer concentrations (10, 5, 2.5, 1.25, and 0.625 μM) and template concentrations (ranging from 1.0 × 10⁷ to 1.0 × 10³ CFU/mL). Different colors indicate different primer concentrations. (**B and C**) Amplification and melting curve analyses performed using the S2-2-specific gene-based qPCR assay.

Based on the identified strain-specific region, a pair of qPCR primers named S2-2 was designed. Based on the qPCR amplification curves, clear gradient patterns were observed in response to both template dilution series and gradient primer concentrations, with consistent melt peaks at 79°C (Fig. 4B and C). The results demonstrated that at a primer concentration of 1.25 μM, the amplification efficiency reached a maximum of 101.79%, with the lowest detection limit observed at 1.0 × 10^1^ CFU/mL. At 0.625 μM, the efficiency was 100.51%, with a detection limit of 1.0 × 10^2^ CFU/mL. Higher concentrations (≥2.5 µM) resulted in relatively lower efficiencies with no significant improvement in detection limits (Table 2; Fig. 4A). Specificity testing was conducted against common gut microbiota (*E. coli*, *Staph. aureus*), and additional closely related *Bacillus* strains (*Bacillus subtilis*, *Bacillus megaterium*, *Bacillus cereus*, and *Bacillus licheniformis*) (Fig. S1). Results showed that no amplification was observed in any of the tested non-BS9 strains (Cq > 35), confirming the high specificity of the S2-2 primer pair for *B. subtilis* BS9.

**TABLE 2:** Primer amplification parameters

Primer name	Primer concentration (μM)	Efficiency (%)	*R* ^2^	Detection limit (CFU/mL)
S2-2	10	98.56	1.00	1.0 × 10^1^
	5	98.91	1.00	1.0 × 10^2^
	2.5	98.29	1.00	1.0 × 10^1^
	1.25	101.79	0.99	1.0 × 10^1^
	0.625	100.51	1.00	1.0 × 10^2^

### Gastrointestinal tract persistence distribution

**Fig 5:**
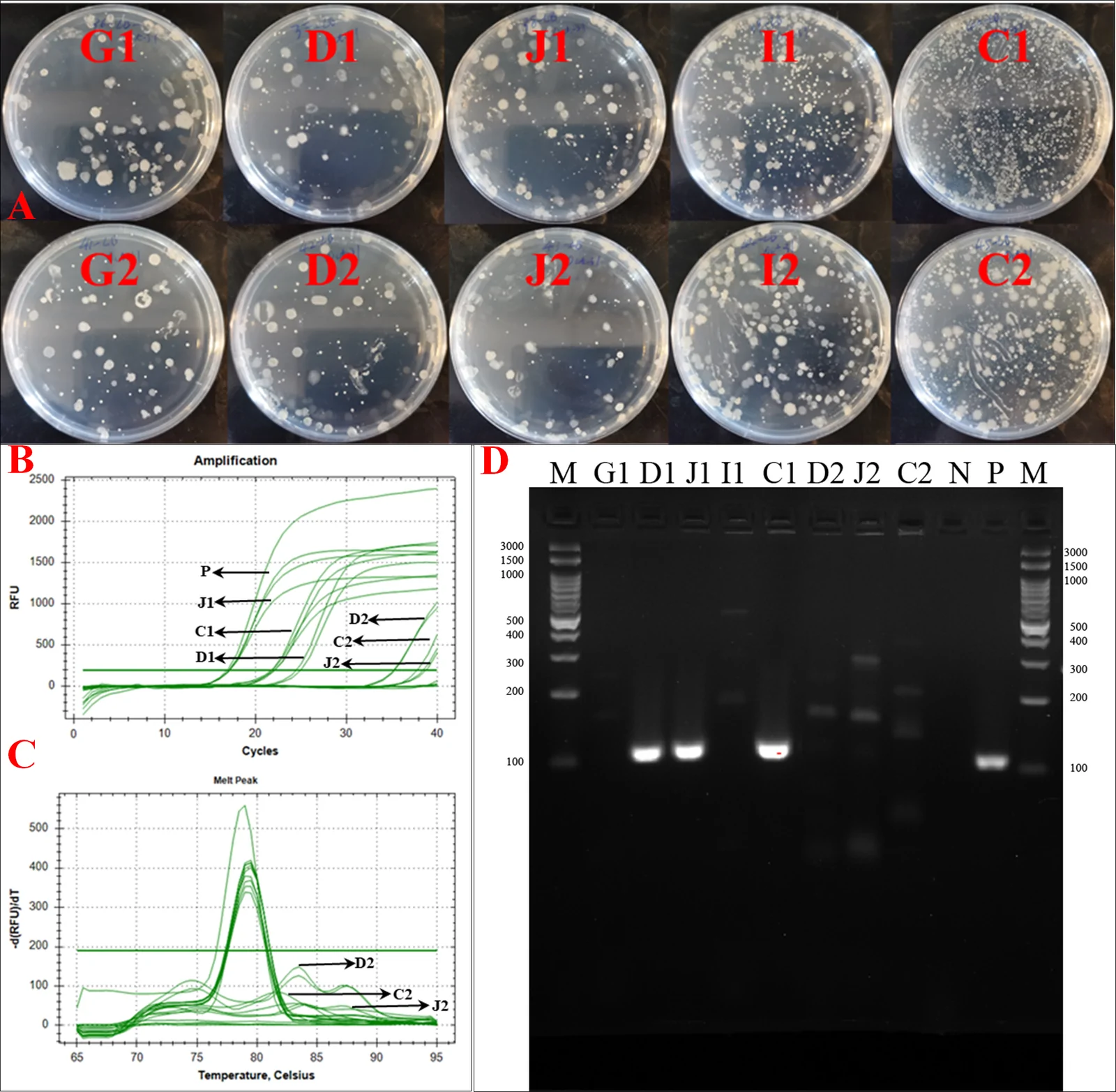
S2-2-specific gene detection of BS9 in chicken intestinal tract. (**A**) According to the results of qPCR, the results of BS9 from intestinal samples were confirmed by plates assay. The different intestinal samples from BS9-supplemented group and control group at day 28 were mixed, and 100 μL of each sample suspension cultured on the LB agar plates at 37°C for 16 h. (**B and C**) Amplification and melting curves of DNA of mixed bacteria samples from the gizzard, duodenum, jejunum, ileum, and ceca in BS9-supplemented group and control group, using S2-2-specific gene qPCR method. (**D**) Electrophoresis on a 1.5% agarose gel of qPCR products obtained from positive samples using S2-2-specific gene qPCR method. M: 100 bp DNA ladder (Invitrogen); G1/G2: gizzard samples from BS9-supplemented group and control group; D1/D2: duodenum samples from BS9-supplemented group and control group; J1/J2: jejunum samples from BS9-supplemented group and control group; I1/I2: ileum samples from BS9-supplemented group and control group; C1/C2: ceca samples from BS9-supplemented group and control group; N: nuclease-free water as negative control; P: genome DNA of BS9 as positive control.

The collected specimens from six birds per group were screened and identified using the *B. subtilis* BS9 strain-specific qPCR assay. Both Pool A and Pool B from the duodenum, jejunum, and cecum from the BS9-supplemented group yielded positive amplification (Cq ≤ 35) in all three technical replicates, whereas both pools from the gizzard and ileum were consistently negative; all pools and segments from the control group were negative (Fig. S2). To further confirm these results, single colonies were picked (Fig. 5A) and subjected to strain-specific qPCR. The qPCR products were analyzed by agarose gel electrophoresis. Results showed a target band of the expected size (110 bp) was again evident in the pooled samples from the duodenum, jejunum, cecum, respectively, and the positive control (Fig. 5D). Clear amplification curves were observed with a characteristic melting peak at 79°C (Fig. 5B and C), further verifying BS9 was indeed colonizing in these intestinal sites. The direct qPCR results from the pooled samples were fully consistent with the colony qPCR results, confirming the reliability of the detection (Table S2).

## DISCUSSION

**Fig 6:**
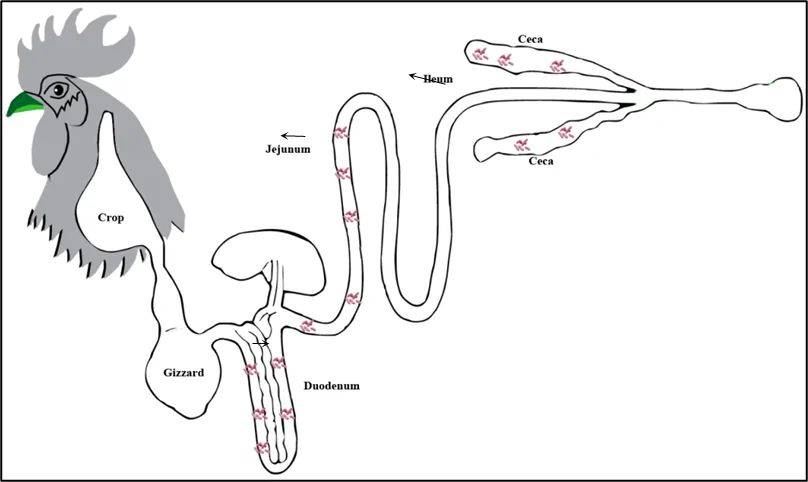
Colonization of *B. subtilis* BS9 in different segments of the broiler gastrointestinal tract.

Our study delineates a clear spatial persistence pattern of *B. subtilis* BS9 in the broiler gastrointestinal tract, with successful detection in the duodenum, jejunum, and cecum, but not in the gizzard or ileum (Fig. 6). This preferential detection is crucial for understanding how BS9 interacts with the host in specific physiological niches to exert its probiotic functions. Many beneficial effects of probiotics, such as immune modulation and barrier enhancement, are triggered by their localized presence at key functional sites, rather than overall high abundance. The detectable presence of BS9 at 14 days post-administration, acknowledging that our single time-point sampling captures a snapshot rather than dynamic colonization processes. The duodenum represents a relatively challenging environment for bacterial colonization among intestinal segments. Incoming bacteria must first endure stresses from gastric acid, bile salts, and digestive enzymes (14), in addition to the rapid peristaltic movement in the duodenum, which limits prolonged retention (15). However, certain highly resilient microorganisms, however, are notable for their ability to adapt to these harsh conditions. Notable examples include lactic acid bacteria (16), yeast (17), and spore-forming bacilli (*B. subtilis*, *B. licheniformis*, and *B. coagulans*) (18). Bacterial spores exhibit strong resistance to gastric acid, bile salts, and high temperatures. As a strain of *B. subtilis*, BS9 can form highly specialized endospores under nutrient deprivation and other stressful conditions, thereby resisting duodenal environmental pressures and achieving detectable colonization. Ruan et al. (19) demonstrated via reverse transcription-PCR that orally administered *B. subtilis* spores can survive stresses such as gastric acid in the upper gastrointestinal tract through the expression of sporulation-specific genes. This offers a plausible explanation for detection of *B. subtilis* BS9 in the duodenum 14 days after supplementation stopped. In contrast to the duodenum, *B. subtilis* BS9 was undetectable in the gizzard. We speculate that, in addition to the similarly low pH environment as in the duodenum, prolonged intermittent mechanical abrasion is the primary cause, particularly after 14 days of administration. This mechanical stress is likely sufficient to disrupt *B. subtilis* BS9 viability.

Bile salt tolerance is a prerequisite for the intestinal colonization of *Bacillus subtilis*. Strains with poor bile resistance undergo significant mortality during this process, resulting in an insufficient number of viable cells reaching the target sites and failing to establish effective colonization (14). In contrast, strains with strong bile tolerance maintain their germination potential even in the presence of bile. Once they reach regions with lower bile concentration, such as the mid-to-distal small intestine or the cecum, where they can rapidly germinate and secure an ecological niche, initiating the colonization process (14). Moreover, bile-adapted strains sustain normal metabolic activity under such conditions, continuously secreting digestive enzymes and antimicrobial substances, like bacteriocins and surfactin. This functional activity enables them to fulfill probiotic roles, such as aiding digestion and inhibiting pathogens (20). Therefore, the ability of *B. subtilis* BS9 to colonize the duodenum reflects its bile tolerance and provides a foundational basis for exerting probiotic effects in subsequent intestinal segments. Our previous work on the tolerance of BS9 on bile salt *in vitro* is consistent with this notion (5). This feature allows positioning this probiotic at the entrance of the intestinal tract, enabling it to act as a first line of defense against incoming pathogens like ETEC through competitive exclusion and its documented contact inhibition mechanism. Furthermore, its survival in this harsh environment provides a reservoir of viable cells that can continuously seed the downstream intestinal segments, ensuring sustained probiotic activity.

The cecum can be described as a specialized fermentation chamber, providing an almost ideal environment for the germination and growth of facultative anaerobic spore-forming bacteria such as *B. subtilis* BS9. It is rich in nutrients, including undigested proteins, carbohydrates, and lipids, which can support spore germination and subsequent vegetative growth (21). Moreover, the cecum is characterized by an anaerobic environment. As a facultative anaerobe, *B. subtilis* BS9 can perform both aerobic respiration and anaerobic fermentation, granting it a competitive advantage over strict anaerobes in this niche (22). The extended retention time of cecal contents further supports spore germination and bacterial proliferation. Additionally, the near-neutral pH within the cecum helps relieve inhibition of spore germination.

Compared with the duodenum, the mid-intestinal tract presents a less hostile environment where bile and pH are no longer the primary constraints, significantly improving the survival probability of probiotics. This region serves as the major site for nutrient absorption, abundant with readily absorbable small molecules, such as oligopeptides, oligosaccharides, amino acids, and monosaccharides, which are resulting from enzymatic digestion (13, 23). The retention time in this segment is longer than in the duodenum but shorter than in the cecum, which is sufficient for probiotics with adhesion capabilities to initiate the “recognition–adhesion–colonization” process yet remains inadequate for all strains to succeed. This may explain why *B. subtilis* BS9 was detected in the jejunum but not in the ileum; that is, a discrepancy potentially attributable to the transit rate of digesta, which likely prevented effective colonization in the ileum. The microaerobic conditions provide a competitive advantage for facultative anaerobes such as *Bacillus subtilis* and *Lactobacillus* (24). Importantly, the mid-intestine features a vast absorptive surface area (villi and microvilli) covered by a thick mucus layer (25, 26), which offers adhesion sites for probiotics (27). This aligns with our earlier emphasis on the significance of high-sensitivity detection methods for capturing low-abundance colonization. In support of these observations, Casula et al. (28) targeted a genetically engineered chimeric gene ftsH-lacZ, which is selectively and strongly expressed in vegetative cells of *Bacillus subtilis*. Their findings indicated extensive spore germination in the jejunum and ileum, consistent with our results.

The strain-specific qPCR method provides a necessary tool for tracking probiotic persistence, while the identified preferential distribution pattern of BS9 lays a foundation for understanding its probiotic mechanisms and application in antibiotic-free broiler production. However, a limitation of the current work is that sampling was performed only at a single time point (14 days after the supplementation completed), which does not fully capture the dynamic persistence process of *B. subtilis* BS9 in the host intestine. Furthermore, while the qPCR assay was developed as a quantitative method, its *in vivo* application was qualitative presence or absence. Future studies should apply the quantitative capability of the assay to determine bacterial loads in different intestinal segments and should include multiple time points and integrate transomics or metabolomic approaches to better elucidate the spatiotemporal relationship between colonization patterns and probiotic effects. Another limitation of this study is the pooling strategy used for colonization assessment. Samples from six birds per group were pooled prior to analysis to enable efficient screening, consequently, the detection outcome represents the presence of BS9 in the pooled population rather than individual-level prevalence. Additionally, the pooling strategy generated only two independent samples (Pool A and Pool B) per segment per group for direct qPCR, precluding robust statistical comparisons between groups. Despite this limitation, the stringent dual-pool and dual-assay approach requiring both pools to be positive by qPCR with confirmation by colony qPCR, provides strong evidence for the region-specific persistence of BS9. Future studies with individual-level sampling and larger sample sizes are warranted to quantify the true prevalence and enable robust statistical inference.

## Data Availability

The data that support the findings of this study are available from the corresponding author. The sequencing data generated in this study have been deposited in the Sequence Read Archive (SRA) under the BioProject accession number PRJNA1406684.

## SUPPLEMENTAL MATERIAL

Supplemental tables and figures spectrum.00926-26-s0001.pdf Tables S1 and S2; Fig. S1 and S2.

ASM does not own the copyrights to Supplemental Material that may be linked to, or accessed through, an article. The authors have granted ASM a non-exclusive, world-wide license to publish the Supplemental Material files. Please contact the corresponding author directly for reuse.
