# Supplementary material for: Genome-informed qPCR tracking revealed preferential persistence of Bacillus subtilis BS9 in the broiler chicken gastrointestinal tract
Source: Microbiol Spectr. 2026 Sep 17;14(10):e00926-26. doi: 10.1128/spectrum.00926-26 (PMC13688835; doi:10.1128/spectrum.00926-26)
Supplement: Supplementary file 1 [file spectrum.00926-26-s0001.pdf]

Supplementary Table 1

Supplementary Table 1.
Primers for *B. subtilis* BS9 strain-specific gene detection

Gene	Product size	Forward 5’-3’	Reverse 5’-3’	Annealing Tm
S2-2	110	ATCAGCCGATCCGTTGTTGA	CGCTGTTAATTTCAGCCGGT	60°C

Supplementary Table 2

Supplementary Table 2.
Detection of *B. subtilis* BS9 in different intestinal segments based on pooled samples from six birds per group

Intestinal segment	Direct qPCR (Pool A / Pool B)[1]	Colony qPCR (positive/10 colonies)[2]	Control group (direct qPCR + colony qPCR)[3]
Gizzard	−/−	0/10	−/−, 0/10
Duodenum	+/+	**4/10**	−/−, 0/10
Jejunum	+/+	**2/10**	−/−, 0/10
Ileum	−/−	0/10	−/−, 0/10
Cecum	+/+	**3/10**	−/−, 0/10

[1] For each intestinal segment, samples 1–3 from six birds were pooled as Pool A, and samples 4–6 were pooled as Pool B. Each pool was tested in three technical replicates by qPCR. "+" indicates positive amplification in all three technical replicates ($Cq \leq 35$); "−" indicates negative in all three technical replicates.
[2] Ten single colonies were randomly picked from the total mixed plate (all six samples pooled) for each segment and tested by strain-specific qPCR.
[3] Control group (n=6 birds, pooled identically) showed negative results in all segments by both direct qPCR (−/− for both pools) and colony qPCR (0/10 for all segments).

Supplementary Figure 1

Supplementary Figure 1. S2-2-specific gene detection of common gut microbiota and additional closely related *Bacillus* strains

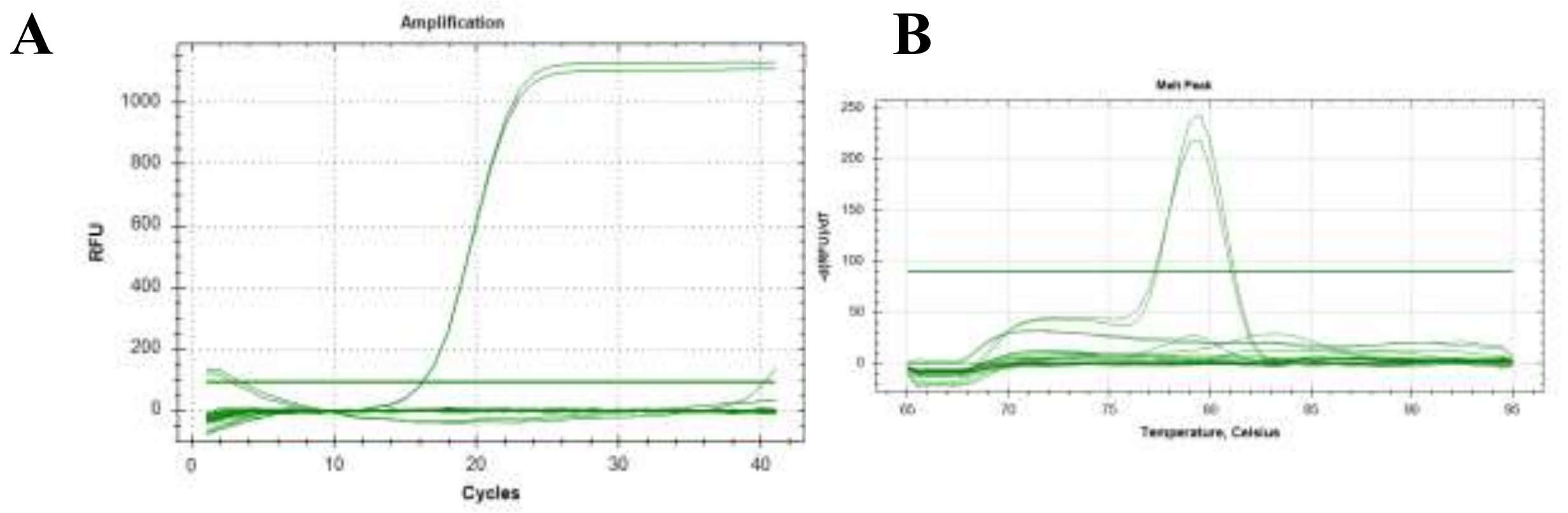

(A, B) Amplification and melt curves of DNA of *Escherichia coli* ATCC 25922, *Staphylococcus aureus* ATCC 6538, *Bacillus subtilis* ATCC 6051, *Bacillus subtilis* strain 168, *Bacillus megaterium*, L1 (*Bacillus subtilis*), L2 (*Bacillus cereus*), L5 (*Bacillus subtilis*), B4 (*Bacillus licheniformis*), B5 (*Bacillus subtilis*) , using S2-2-specific gene qPCR method.

Supplementary Figure 2

Supplementary Figure 2. S2-2-specific gene detection of BS9 in chicken intestinal tract

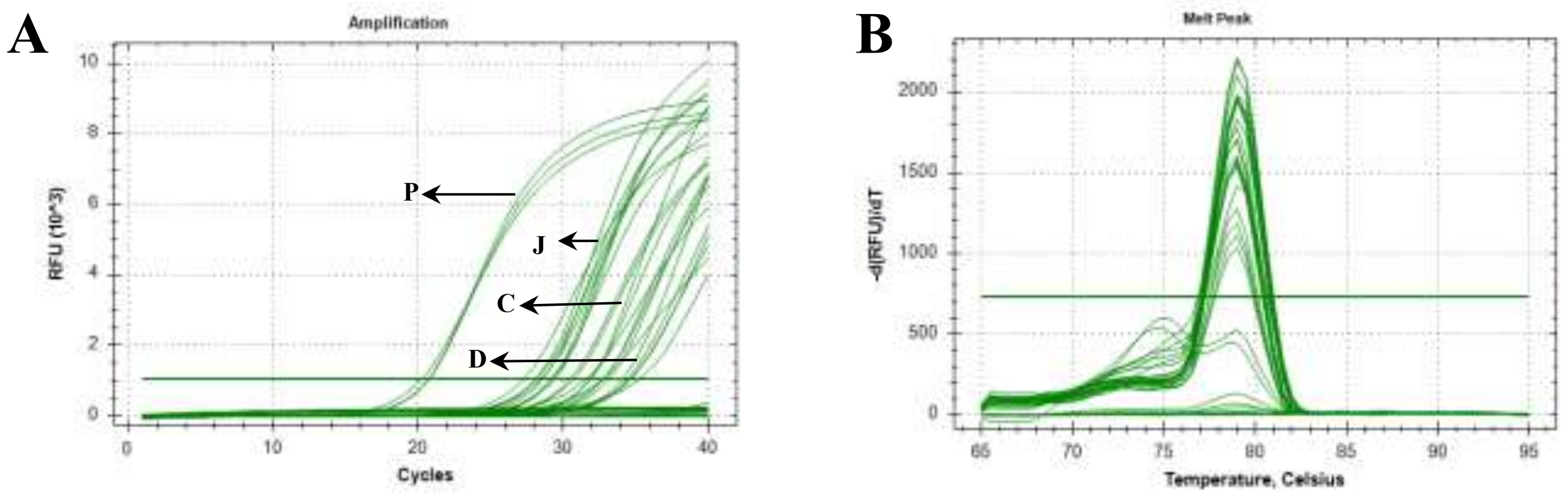

(A, B) Amplification and melt curves of DNA from pooled tissue samples (Pool A and Pool B) from gizzard, duodenum, jejunum, ileum, and ceca from the BS9-supplemented group, using the S2-2-specific gene qPCR method. Pool A: samples 1–3 from six birds were pooled. Pool B: samples 4–6 from six birds were pooled. Each pool was tested in three technical replicates.

D: Duodenum samples. J: Jejunum samples. C: Cecum samples. P: genomic DNA of BS9 as positive control. Both Pool A and Pool B from duodenum, jejunum, and cecum showed positive amplification (Cq ≤ 35) in all three technical replicates, whereas both pools from gizzard and ileum were negative.
